# Evaluating the Impact of Indonesia’s National School Feeding Program (ProGAS) on Children’s Nutrition and Learning Environment: A Mixed-Methods Approach

**DOI:** 10.3390/nu17223575

**Published:** 2025-11-15

**Authors:** Indriya Laras Pramesthi, Luh Ade Ari Wiradnyani, Roselynne Anggraini, Judhiastuty Februhartanty, Wowon Widaryat, Bambang Hadi Waluyo, Agung Tri Wahyunto, Muchtaruddin Mansyur, Umi Fahmida

**Affiliations:** 1 Southeast Asian Ministers of Education Organization Regional Centre for Food and Nutrition (SEAMEO RECFON), Pusat Kajian Gizi Regional (PKGR) Universitas Indonesia, Jakarta 13120, Indonesia; ipramesthi@seameo-recfon.org (I.L.P.); jfebruhartanty@seameo-recfon.org (J.F.); umifahmida@gmail.com (U.F.); 2 Department of Nutrition, Faculty of Medicine, Universitas Indonesia–Dr. Cipto Mangunkusumo General Hospital, Jakarta 10430, Indonesia; 3 Department of Nutrition, Faculty of Sports and Health Sciences, Universitas Negeri Surabaya, Surabaya 60231, Indonesia; lynne.prigel@gmail.com; 4 Directorate General of Primary and Secondary Education, Ministry of Education and Culture Republic of Indonesia, Jakarta 10270, Indonesia; yb1bml@gmail.com (W.W.); bhadiwaluya@gmail.com (B.H.W.); agungtriwahyunto@gmail.com (A.T.W.); 5 Department of Community Medicine, Faculty of Medicine, Universitas Indonesia, Jakarta 10310, Indonesia; muchtaruddin.mansyur@gmail.com

**Keywords:** school feeding program, ProGAS, breakfast, primary school students, school nutrition, impact evaluation, Indonesia

## Abstract

**Background:** Nutrition problems among primary school children increase the risk of illness, reduce school attendance, and impair academic performance. The Indonesian national school feeding program (ProGAS—Program Gizi Anak Sekolah) was developed to address these issues through the provision of healthy breakfast, nutrition education, and character building. **Methods:** This study employed a mixed-methods design involving 454 primary school students aged 8–14 years from 24 schools across four provinces. Data collection included structured questionnaires, 24 h dietary recalls, and anthropometric measurements. In-depth interviews with school principals, teachers, cooking teams, parents, students, nutritionists, and district education office staff were conducted to capture experiences and opinions on the ProGAS implementation. **Results:** ProGAS significantly improved students’ dietary diversity, meal frequency, handwashing with soap, and nutrition knowledge, alongside a positive trend in school attendance. Improvements included higher mean dietary diversity, increased proportion of students eating ≥3 meals/day, greater correct responses on balanced nutrition, and higher rates of handwashing before meals (all *p* < 0.01). Children also developed positive behaviors such as praying before meals, queuing, and taking responsibility for cleaning dishes. However, students’ breakfast habits did not significantly improve, the delivery of nutrition education was suboptimal, and no significant changes were observed in nutrient intakes or nutritional status based on BMI-for-age. While the energy and protein contributions of the ProGAS menu met the recommended 25–30% of daily requirements for breakfast, its micronutrient contributions remained below the recommended levels. Key management gaps include the delivery of nutrition education to students and the monitoring of implementation by local and national authorities. **Conclusions:** ProGAS demonstrated positive impacts on some dietary and hygiene practices as well as learning environment. To achieve greater improvements in breakfast habits, nutrient intake and nutritional status, it is recommended to strengthen the school feeding menus not only for dietary diversity but also for nutrient density, enhance capacity building for teachers, deliver regular and engaging nutrition education, and reinforce program monitoring.

## 1. Introduction

Ensuring adequate nutrition during childhood is essential for supporting growth, cognitive development, school attendance, and long-term human capital formation. Globally, malnutrition among school-aged children remains a pressing issue. Reviews across multiple low- and middle-income countries show that school-aged children often experience stunting, thinness, anemia, and an increasing prevalence of overweight and obesity, reflecting the double burden of malnutrition [1,2]. According to the World Health Organization (WHO), overweight among school-aged children and adolescents has risen significantly in recent decades, posing long-term health risks [3]. School feeding programs have therefore emerged as one of the largest social safety nets worldwide, reaching more than 388 million children in 161 countries prior to the COVID-19 pandemic [4]. Evidence shows that such programs improve dietary intake, reduce short-term hunger, promote school participation, and contribute to better educational outcomes [5,6]. Beyond nutrition, school-based interventions also provide a platform to promote hygiene, social interaction, and character development [7].

The nutrition problems among Indonesian school-aged children have remained a significant public health concern over the past decade. According to the National Basic Health Survey (Riskesdas) in 2013, 30.7% of children aged 5–12 years were stunted, 11.2% wasted, and 26.4% anemic [8]. Although some improvements were observed in subsequent surveys, the prevalence of undernutrition and anemia remained high. In 2018, Riskesdas recorded 30.8% stunting, 10.2% wasting, and 8.0% overweight, with anemia still widespread [9]. The most recent Indonesia Health Survey (SKI) 2023 reported declines in some indicators—21.6% stunting, 7.7% wasting, 10.5% overweight, and 11.2% anemia—among school-aged children [10]. Despite these decreased trends, the prevalence levels continue to exceed the World Health Organization (WHO) thresholds for public health significance: stunting (≥20%), wasting (≥5%), overweight (≥5%), and anemia (≥5%) [3]. This indicates that the double burden of malnutrition, both undernutrition and overweight, remains highly relevant for Indonesia’s school-aged population.

School-aged children often experience inadequate dietary diversity, insufficient micronutrient intake, and poor breakfast habits. Data from national dietary surveys have shown that nearly one-third of primary school children did not meet adequate energy and protein requirements [11]. Breakfast skipping is particularly concerning, as it is associated with poor concentration, fatigue, and increased consumption of unhealthy snacks [12].

In response to these challenges, the Indonesian government has experimented with several school feeding initiatives. The Supplementary Feeding for School Children (Program Makanan Tambahan Anak Sekolah—PMT-AS) was first introduced in 1991 as part of a national poverty alleviation strategy, later decentralized in 2002, and expanded to preschoolers in 2010–2011. In 2016, the Ministry of Education and Culture re-initiated the program as the Nutrition for School Children Program (Program Gizi Anak Sekolah—ProGAS), targeting food-insecure areas [13]. Unlike earlier versions that provided snacks, ProGAS offered complete meals in response to evidence of inadequate energy and protein intake among primary school-aged children. Indonesia also faced measurable food insecurity in 2017, with the prevalence of moderate or severe food insecurity estimated at 8.66% that year. In the rural districts where ProGAS was implemented (Jayapura, Southeast Maluku, Sorong, and West Manggarai), access to adequate and diverse food remained a critical concern, reinforcing the relevance of these areas as priority locations for ProGAS implementation. ProGAS was designed around three integrated pillars: (1) providing nutritious breakfasts to improve dietary intake, (2) nutrition education to increase knowledge and awareness of balanced nutrition, and (3) character building and personal hygiene practices among primary school-aged children [14].

Previous evidence has shown that well-designed school feeding programs can improve dietary diversity, school attendance, and learning outcomes, while also contributing to reductions in hunger and undernutrition [5,15]. However, evaluations of large-scale government-led school feeding initiatives in low- and middle-income countries, including Indonesia, remain limited. Using both quantitative and qualitative methods, this study offers broader insights into the outcomes and processes of school nutrition programs, contributing valuable evidence to the field. This is particularly important given that Indonesia has recently launched a new Free Nutritious Meals (Makan Bergizi Gratis—MBG) program in 2024 as part of its national policy agenda. Lessons learned from earlier initiatives such as PMT-AS and ProGAS are therefore critical to inform the effective design, scaling, and sustainability of MBG.

This study therefore aims to evaluate the impact of ProGAS on nutrition knowledge, dietary practices, hygiene behaviors, character building, nutritional status, and learning environment of primary school children in Indonesia. In addition, the study also aimed to explore the implementation of ProGAS from the perspectives of various stakeholders through a qualitative approach. Findings from this evaluation are expected to provide valuable lessons for strengthening and expanding the national school feeding program in Indonesia, as well as contributing to the global evidence base on school-based nutrition interventions.

## 2. Materials and Methods

### 2.1. Study Design and Setting

This study employed a quasi-experimental pre–post design without a control group to evaluate the impact of the National School Feeding Program (Program Gizi Anak Sekolah/ProGAS). Data collection was conducted at baseline and endline over the course of 120 feeding days, known as Hari Makan Anak/HMA, or Children’s Meal Day.

Selection of provinces and the districts for ProGAS implementation were based on food security and vulnerability status as outlined in the 2015 Food Security and Vulnerability Atlas of Indonesia, published by the Food Security Council, Ministry of Agriculture, and the World Food Program. In addition, ProGAS targeted districts classified as 3T (remote, disadvantaged, and outermost areas) and those included in the 100 priority districts for stunting reduction determined by the National Team for the Acceleration of Poverty Reduction (TNP2K) in 2017. In 2017, a total of 11 districts from five provinces (East Nusa Tenggara, Maluku, Papua, West Papua, and Banten Provinces) were selected by the Ministry of Education and Culture to receive ProGAS. In total, there were 563 schools of ProGAS beneficiaries in these five provinces.

### 2.2. Study Participants and Sampling Procedures

The sample size was calculated using a paired pre–post intervention formula [16], aiming to detect a minimum change of 0.46 g/dL in hemoglobin levels, with a standard deviation (SD) of 1.47 g/dL [17], a 95% confidence interval, 90% statistical power, and a design effect (DEFF) of 2. This calculation yielded a minimum requirement of 430 students. All fourth-grade students at baseline (*n* = 562) and all fifth-grade students at endline (*n* = 497) from the selected schools were invited to participate. Eligible participants were primary school children who were enrolled in the selected schools, present at both baseline and endline assessments, and in good health at the time of measurement (*n* = 454). The reduction in respondents at endline was primarily due to students transferring to other schools or towns or being absent on the day of data collection. In addition, students, school principals, teachers, parents, cooking committees, district education officers, and community health workers (puskesmas) were engaged in the qualitative component of the study to capture broader perspectives on program implementation.

For the present study, one district in each province outside Java Island (i.e., East Nusa Tenggara, Maluku, Papua, and West Papua) were purposively selected, considering accessibility based on recommendations from provincial and district education offices. In each selected district, one sub-district was randomly chosen. Given the minimum number of samples and districts, a total of 24 schools were included in this study, which were selected proportionally to the total number of schools in the selected sub-district. They were four schools in Jayapura (Papua), five schools in Southeast Maluku (Maluku), five schools in Sorong (West Papua), and nine schools in West Manggarai (East Nusa Tenggara). The schools in each selected sub-district were randomly selected from the list of schools provided by the district education office according to the targeted number of schools in each district.

### 2.3. Intervention: ProGAS Overview and Components

Launched nationally by the Ministry of Education and Culture in 2016, ProGAS prioritized schools in food- and nutrition-insecure districts, including remote islands, border, and post-disaster areas. School selection was coordinated with district education offices based on criteria such as having an active principal and committee, serving primarily low-income families, and not receiving similar assistance [13].

Program components included three pillars: (1) provision of breakfast (≈400–500 kcal and 10–12 g protein per serving, 120 feeding days per year, equivalent to three times per week during the school year), (2) nutrition education on balanced and safe diets, and (3) hygiene promotion (e.g., handwashing with soap) and character education (e.g., praying, queuing, finishing meals, and cleaning dishes). Community participation was emphasized, with local farmers supplying ingredients and women’s groups (PKK) involved in meal preparation. By 2017, ProGAS reached over 100,000 students across 11 districts in five provinces and was further expanded nationwide in subsequent years [13].

To facilitate nutrition education, the ProGAS module was developed collaboratively by the Ministry of Education (MoE), Ministry of Health (MoH), the Indonesian Food and Drug Authority (FDA), IPB University, and SEAMEO RECFON. The module encompasses three main areas: (1) healthy food provision, including menu planning, preparation, processing, serving, food safety, and the prevention and control of foodborne illness; (2) nutrition education, covering the dietary needs of school-aged children and the Guidelines for a Balanced Diet; and (3) character education, focusing on hygiene promotion (e.g., handwashing with soap) and practical activities such as establishing fish ponds and school gardens [13,14]. SEAMEO RECFON, which supported the program’s development, also conducted its evaluation.

### 2.4. Data Collection

In general, three pillars of ProGAS (i.e., healthy breakfast, nutrition education and character building) and the program implementation were evaluated using structured questionnaire, observational checklist, and in-depth interviews. The program impacts were examined through anthropometric, hemoglobin, and dietary intake assessments.

#### 2.4.1. Student Characteristics, Nutrition Knowledge, Character Building and Personal Hygiene Practices, and Learning Environments

The data collection was conducted through interviews to the students using a pre-tested structured questionnaire which measured student characteristics (sex, age, parental education and occupation, number of siblings). It also assessed balanced nutrition knowledge (dietary diversity, clean and healthy lifestyle, weight monitoring, benefits of breakfast) and balanced nutrition practices (meal frequency, breakfast habits, water intake, snacking behavior). The questionnaire further measured personal hygiene practices (handwashing with soap, toothbrushing, bathing), character building aspects (praying before class and meals, queuing, finishing meals), morbidity history (diarrhea, common cold, cough, fever), and learning environment outcomes (feeling hungry, sleepy, comfortable). Nail and hair cleanliness were assessed through direct observation.

#### 2.4.2. Dietary Intake and Practices

A single 24 h dietary recall was conducted for all students, with repeated recalls administered on non-consecutive days to a 40 subsample. Nutrient intake was analyzed using Nutrisurvey for Windows 2007 and the Indonesian food composition database. Prior to analysis, energy under- or over- reporting was assessed by comparing the reported energy intake (rEI) with energy requirements estimated by using total energy expenditure predicted (pTEE) [18]. No over-reporting was found, while 33 students (6.7% of the sample), mostly male (*n* = 24), were identified as under-reporters. For evaluating ProGAS impact on nutrient intake, only students with complete dietary data at both baseline and endline and who continued consuming the ProGAS breakfast menu at endline were included, resulting in a final sample of 51 students for paired analysis.

Referring to the FAO guidelines for measuring household and individual dietary diversity, the dietary diversity score (DDS; range 0–7) was calculated by summing the consumption of seven food groups: (1) carbohydrate sources (grains, tubers), (2) legumes, (3) milk and dairy products, (4) meat, poultry, fish, seafood, liver, and other organ meats, (5) eggs, (6) vitamin A-rich fruits and vegetables (dark green leafy vegetables, yellow/orange vegetables, yellow fruits), and (7) other fruits and vegetables [19]. DDS was derived from the first 24 h dietary recall.

#### 2.4.3. Nutritional Status

Two measurements of students’ weight and height were recorded to nearest 0.1 kg and 0.1 cm, respectively. The average of two values was used for analysis. Body weight was measured using a calibrated SECA 770 weighing scale, and height was measured using a Shorr Board, following standard WHO procedures. Nutritional status was defined as a BMI-for-age Z-score based on the WHO growth standards [20]. Hemoglobin level was assessed from capillary blood (drawn with finger prick) using HemoCue Hb 201+ device.

#### 2.4.4. Qualitative Data

In-depth interviews were conducted to explore stakeholders’ experiences and perceptions regarding the implementation of ProGAS. A total of 125 participants were involved, including district education (4) and health officials (4), school principals (24), teachers (24), parents (18), cooking staff (13), and students (38). Both interviews and observations were carried out by trained field supervisors using structured question guides.

### 2.5. Data Analysis

Quantitative data were entered and cleaned using double entry in SPSS for Windows Version 21.0. Data accuracy was ensured by checking for missing values, outliers, and consistency. Normality was tested using the Kolmogorov–Smirnov test (*p* > 0.05). Descriptive statistics were presented as means or medians for continuous variables and frequencies or percentages for categorical variables. Pre–post comparisons were conducted using *t*-tests or McNemar tests as appropriate.

Qualitative data analysis was carried out in two stages. In the field, interview responses were summarized into respondent-specific matrices based on the variable indicator matrix (VIM) to ensure completeness and capture initial patterns. A desk-based analysis then refined these findings, guided by the study objectives, to verify the summaries and provide deeper explanations of emerging themes and patterns. Analysis of the qualitative data was guided by five pre-determined themes, including program implementation, program monitoring, beneficiary and implementer acceptance and feedback, challenges encountered by implementers and stakeholders, and the perceived benefits of the program on students’ dietary practices. During analysis, two additional themes emerged and were subsequently incorporated: students’ behaviors related to finishing the ProGAS meals and teachers’ responses to these behaviors, as well as variations in guidelines and practices across districts and health centers when modifying less-preferred ProGAS menus.

For presentation purposes, however, Section 3 was organized in accordance with the three main pillars of ProGAS, namely improving nutritional intake through school breakfasts (Pillar 1), nutrition education (Pillar 2), and character building and personal hygiene (Pillar 3), with the identified themes integrated into the relevant pillars.

## 3. Results

### 3.1. Student Characteristics

The following analysis presents the characteristics of the students who participated in both baseline and endline surveys (454 students) (Table 1).

### 3.2. Pillar 1: Improved Nutritional Intake Through School Breakfast

#### 3.2.1. Dietary Diversity and Practices

Based on a single 24 h dietary recall, the mean DDS increased significantly from 3 to 4 food groups consumed (*p* < 0.001). During the program, students reported higher consumption of fruits, fish, and eggs (Table 2).

The proportion of students consuming at least three meals per day increased significantly by 5.8% (*p* = 0.007); nevertheless, 14.3% still reported consuming fewer than three meals daily. In contrast, only a slight, non-significant improvement was observed in daily breakfast habits: 58.8% of students reported having breakfast every day at baseline compared to 61.2% at endline, while 38.8% still did not regularly consume breakfast at endline. This suggests that outside of the designated Hari Makan Anak (Children’s Meal Day), many students did not consistently have breakfast at home, indicating that PROGAS alone was insufficient to fully optimize breakfast habits.

School breakfast provision was also associated with changes in students’ snacking behavior. A significant reduction was noted in the proportion of students bringing pocket money and purchasing snacks at school. Parents corroborated this observation, reporting that they were less likely to provide pocket money since the school breakfast was considered sufficient to prevent hunger during school hours. Consistent with this, consumption of snacks (cakes/pastries/bread) declined from 53.9% at baseline to 44.2% at endline, and packaged beverage consumption decreased from 46.6% to 38.6%.

#### 3.2.2. Dietary Intake

The study shows that students’ median nutrient intake, both before and after ProGAS implementation, falls short of their daily nutritional requirements. While there was a trend of increasing intakes for energy, carbohydrates, protein, fat and micronutrients (vitamin A, vitamin B6, calcium, iron, and zinc), the increase was not statistically significant (Table 3).

#### 3.2.3. Contribution of ProGAS Breakfast

During the endline assessment, most schools had already completed the implementation of the breakfast activity. From the few schools (5 out of 24 schools) that were still carrying out this activity at the time of data collection, interview data on breakfast consumption provided by ProGAS were successfully collected and analyzed. The following table shows the contribution of ProGAS breakfasts consumed by 51 student respondents (Table 4).

The percentage contributions of energy and nutrients from the ProGAS meal consumed by children were below the recommended levels, particularly for fat and micronutrients. Carbohydrates showed the highest contribution (20.3%), while calcium was the lowest (1.5%).

Data shows that 26.7% of students did not always finish the ProGAS breakfast provided. The reasons cited included disliking the menu (48.8%), the portion size being too large (36.4%), and already having had breakfast at home (14.7%). Qualitative exploration through student and teachers interviews revealed that the least preferred menus, due to taste or texture, were root-based porridge, traditional rice porridge with vegetables and sweet corn, green bean porridge, steamed sweet potatoes, kolak (a sweet dessert made with banana, sweet potato, or pumpkin cooked in coconut milk and palm sugar), and melon. In contrast, dishes such as noodles, eggs, chicken, watermelon, orange and papaya were consistently finished by students. Interestingly, students mentioned that they and their peers were allowed to request rice portions according to their preference from teachers, which often enabled them to finish their meals more easily.

*“Our teacher told us that if we weren’t hungry, we could just ask for half a portion of rice, and later we could ask for more (if we want).”*

*(female student, Sorong)*

If students did not finish their meals, teachers usually reminded them and asked them to eat all of it (at least the side dishes). In some cases, sanctions were given, such as requiring students to clean the classroom for a week or not allowing them to leave the classroom until the food was finished. Teachers also conveyed that finishing the meal was a way of expressing gratitude to the cooks. Some students reported that they always finished the ProGAS breakfast because they believed the meal is important for their growth.

*“The teacher reprimanded us and usually told us to finish (the meal) until the plate was clean.”*

*(male student)*

*“The teacher told us we had to finish the food because the cooks worked hard to prepare it.”*

*(female student)*

*“We have to finish the meal because it is healthy. If we don’t finish it, we can’t be tall”.*

*(female student)*

However, not all teachers required students to finish their meals, including when students were unwell or stated that they were unable to do so. In such situations, students reported that teachers did not reprimand them for leaving food unfinished.

*“He (the teacher) said ‘just leave the plate (with the unfinished meal) there”*

*(male student)*

*“We were never told to finish the food, nothing happened because the teacher didn’t know.”*

*(male student)*

An additional perspective highlighted by teachers was that the food provided through the program did not contain MSG, whereas children were generally accustomed to more savory meals at home. Teachers also suggested that unfinished meals could be attributed to students already being full of breakfast consumed at home or from snacks purchased at the school canteen, particularly in schools where the ProGAS breakfast was served during the first class break, around 9 am (reported by 61.7% students) or 10 am (reported by 27.1% students). Teachers shared their efforts to persuade the students to finish the meal.

*“We have already explained to them (the students) that the food has been prepared and must be finished. The habit of finishing food should also be applied at home.”*

*(male teacher)*

*“I always tell them it is a pity to waste food since it is a blessing and because someone has put effort into cooking it. Sometimes children’s motivation to eat increases if we create a competition, such as rewarding those who finish the meal the fastest.”*

*(female teacher)*

### 3.3. Pillar 2: Nutrition Education

At endline, 66.1% of students correctly answered 12 out of 15 questions on balanced nutrition, representing a significant 15.5% increase from baseline (*p* < 0.001). The greatest improvements were observed in knowledge of examples of appropriate foods for breakfast (+23%) and personal hygiene, particularly handwashing with soap (+18.1%). However, knowledge about the causes of obesity remained low, with only 30–36% of students correctly identifying that the statement “Eating three times a day causes obesity” is incorrect (Table 5).

Although nutrition education is one of the three main components of ProGAS, about 22% of students reported that they did not or rarely receive nutrition education from their teachers. Among students who did receive it, the most frequently recalled messages were the importance of finishing the ProGAS breakfast to stay healthy (34.5%), washing hands with soap before eating (22.8%), and praying before meals (12.2%). Smaller proportions recalled messages about eating nutritious foods to maintain health (8.7%) and being disciplined during the ProGAS breakfast session (8.4%). Only a very small proportion mentioned brushing teeth after meals and eating fruit frequently.

According to program guidelines, nutrition education in ProGAS should be delivered by teachers during breakfast sessions or other relevant occasions. When explored further through in-depth interviews, students and teachers reported that nutrition messages were usually delivered orally by teachers, without specific educational materials, either before or during the ProGAS breakfast sessions, during break time, in class (typically during science lessons), during school duties, religious activities, or cleaning-the-school activities. Although rarely implemented, the nutrition messages were also delivered through storytelling, singing, and drawing.

Almost all students mentioned that they never asked questions during these nutrition education sessions. Among the few who did, the questions were related to the nutritional content of fruits, examples of nutritious foods, and personal hygiene.

*“Never, because we don’t get used to it (asking question). Actually, I wanted to ask, but I was too shy.”*

*(female student)*

Teachers acknowledged facing several challenges in delivering nutrition education to students, including the limited availability of age-appropriate learning materials, the diverse socio-economic backgrounds of students that made tailoring nutrition messages difficult, the tight school schedule, and teachers perceived lack of sufficient nutrition knowledge. Similarly, nutritionists from the health center were also challenged by their tight schedule in providing services to target beneficiaries. Thus, they could only provide education with ‘generic message’ such as washing hands before eating.

*“I am responsible for many posyandu (a community-based health post that provides health and nutrition services for pregnant women and young children), so I have not been able to provide much education specifically for ProGAS. But before meals, I usually give counseling, such as handwashing before eating. However, in one school I only cover three classes at a time, and on the next visit I rotate to other classes.”*

*(Nutritionist)*

### 3.4. Pillar 3: Character Building and Personal Hygiene Practices

Consuming plain water is one of the key recommendations in the Indonesian Balanced Nutrition Guidelines. ProGAS required every student to bring drinking water from home, which resulted in a significant increase in the proportion of students bringing water bottles to school (from 39.2% at baseline to 45.1% at endline, *p* < 0.001). This practice was associated with an increase in mean daily water consumption, from 723 mL at baseline to 840 mL at endline (Paired *t*-test, *p* = 0.028). However, this amount remained below the recommended daily water intake for children aged 10–12 years, which is 1800 mL according to the Indonesian Recommended Nutrient Intakes (2013).

Character building activities were also integrated into the ProGAS activities, including praying (before class, and before and after meals), queuing, and finishing meals. These practices were reinforced through daily school activities and showed significant improvement at endline (Table 6). Observations during the endline survey revealed that students eagerly took initiative in leading prayers, lining up for handwashing and meal distribution, and joyfully finishing the provided school breakfast.

Majority of the students washed their hands with soap before eating (96.2%). Several schools that suffered from water shortage instructed older students (grade 4-5-6) to bring a bucket of water for washing hands and dishes. Buckets with lid and tap were used by those schools without tap water. Soap was commonly shared between classes. Cleanliness of nails and hair increased by 13.2% (*p* < 0.001) and 10.2% (*p* < 0.001), respectively.

### 3.5. Nutritional Status and Learning Environment Outcomes

#### 3.5.1. Nutritional Status

**Figure 1:**
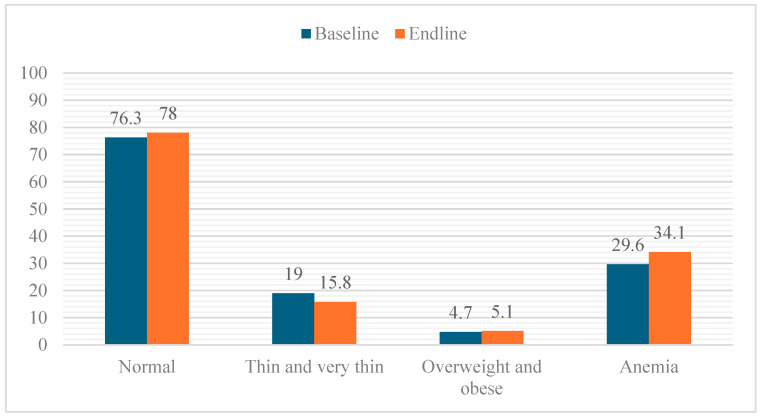
The percentage distribution of students across nutritional status categories at both time points.

No significant change was observed in the mean BMI-for-age z-score (BAZ), which remained relatively stable from baseline (−0.83 ± 1.33) to endline (−0.81 ± 1.39; *p* = 0.498). This finding suggests that overall nutritional status did not improve during the program period. However, the distribution of nutritional status categories showed a modest positive shift: the prevalence of thin/very thin students decreased from 19.0% to 15.8%, while the proportion with normal nutritional status slightly increased (76.3% to 78.0%). The prevalence of overweight and obesity remained low and unchanged (around 5%). In contrast, there was an indication of increased anemia at endline, although the difference was not statistically significant (29.6% to 34.1%; *p* = 0.133). However, the mean hemoglobin level declined significantly from 12.1 ± 1.3 g/dL at baseline to 11.9 ± 1.1 g/dL at endline (Figure 1).

#### 3.5.2. Students’ Learning Environment at School

Students’ learning environment demonstrated meaningful improvements over the course of the program. Most notably, feelings of comfort in class increased substantially, with nearly nine out of ten students reporting being comfortable at endline (*p* < 0.001). At the same time, the proportion of students feeling hungry during class declined significantly (*p* = 0.008), suggesting that the school meals helped reduce hunger during school hours. Other indicators, such as activeness and sleepiness in class, showed no significant change (Table 7).

### 3.6. Perceived Benefits and Challenges of ProGAS Implementation

In addition to the quantifiable impacts on students, the exploration also identified perceived benefits of ProGAS among various stakeholders. One such benefit was the provision of knowledge and skills in preparing healthy meals for children, gained through training sessions for the cooking teams as well as through their experience in preparing ProGAS menus. Most cooking groups reported that their motivation to participate was not driven by financial incentives. Rather, they expressed a willingness to contribute voluntarily to ensure the smooth implementation of the program, particularly because it was seen as beneficial for their children or families, including improvements in hygienic practices and healthy eating at home.

*“At first, I didn’t know there was payment for being part of the ProGAS cooking team. I wanted to join because I thought this program was good for the students. I also have a younger brother who is now in 6th grade.” (female, cooking team)*

The training and experience also encouraged the cooking teams, who were mostly parents, to apply these practices at home. Several respondents reported that they no longer used artificial flavor enhancers in home cooking. Most cooks were also able to demonstrate an understanding of the importance of hygienic practices in food preparation (Table 8).

Interviews with various stakeholders revealed several challenges in the implementation of ProGAS at the study sites. In general, the constraints identified included delayed disbursement of funds, the high cost of food ingredients and equipment, geographical conditions that resulted in long travel times to purchase food, difficulty in balancing teaching duties with program-related tasks (e.g., being assigned to shop for food ingredients), and challenges in managing parents as members of the cooking team, as they often arrived late to school due to household responsibilities. Additional difficulties included limited availability of certain food items required by the recipes (e.g., vermicelli, certain fish) and non-functional cooking equipment. With regard to limited supply of certain food ingredients, nutritionists in some health centers provided alternative menu options which were similar to the main ProGAS menu.

*“Often, during certain seasons, food ingredients such as fish are difficult to obtain, and even if available, the prices are very high. Due to large waves, ships cannot transport food supplies from Ambon (the capital city). Teachers felt grateful for the existence of the district menu, as it helped ease their burden when certain food items were scarce.”*

*(Nutritionist)*

Interestingly, some school principals also highlighted challenges related to anticipating the very high level of parental interest in participating in the program.

*“Many parents wanted to participate, so we had to be careful in communicating with them. We promised that if there were future opportunities, we would rotate their participation. There were also parents who misunderstood, thinking that school fees were being used for food. They questioned why the money was not simply distributed to parents instead.”*

*(School Principal)*

Another challenge concerned monitoring. The District Education Office (DEO) noted the absence of a planned monitoring schedule, despite program guidelines mandating it. Limited transportation funds, coupled with the wide geographic spread of schools, were cited as major constraints. When visits did occur, monitoring mainly focused on verifying the number of feeding days and reviewing school financial reports. The administrative requirements for financial reporting were also considered time-consuming, leaving little room for comprehensive monitoring or proper completion of the official monitoring forms. Some designated monitors were not fully familiar with how to complete these forms. Joint monitoring by the Education Office and the primary health center had never been conducted.

Nutritionists reported procedural barriers in conducting school visits for ProGAS monitoring. Schools were required to issue a formal request letter (or phone request) before visits could take place; without such a request, the nutritionists felt uncomfortable and lacked the basis to conduct monitoring.

During implementation, communication largely relied on WhatsApp groups to track school activities, particularly through photo documentation of breakfast sessions and reporting of feeding days and student numbers. Nevertheless, when resources permitted, education office staff were able to conduct school-visit monitoring.

*“Since the schools under my supervision are located along my route from home to the office, I can visit them every week—about three to four schools each week.”*

*(DEO officer)*

## 4. Discussion

Despite improvements in dietary diversity, this study found that children’s intake of several key nutrients, including macro and micronutrients, remained below EAR to confirm inadequate intake at population level. Consistently low intakes were found based on both the one-day dietary intake (Table 3) and the ProGAS breakfast intake (Table 4).

### 4.1. Pillar 1: Improved Nutritional Intake Through School Breakfast

In 2017, six menu variations were developed for ProGAS. Each menu generally consisted of a carbohydrate source (rice, corn vermicelli, corn porridge, cassava), an animal protein source (chicken, egg, or fish), vegetables, and fruit (such as banana, guava, orange). It should be noted that the ProGAS menu formulation was based on the content of energy (ranged from 415 to 573 kcal) and protein (12–17 g).

Referring to the 2013 Indonesian Recommended Nutrient Intake (RNI), which were in effect during the implementation of ProGAS 2017, our further analysis showed that ProGAS breakfasts contributed, on average, 22.4–31.0% of daily energy requirements, 20.8–28.7% for protein, 16.7–40.1% for carbohydrates, 13.2–45.6% for fat, 2.8–42.8% for vitamin A, 9.0–40.0% for vitamin B1, 10.8–54.2% for vitamin B6, 3.9–44.4% for vitamin B12, 0.9–15.2% for calcium, 6.1–18.2% for iron, and 5.9–16.3% for zinc. This suggests that ProGAS menus met the recommended 25–30% contribution to daily energy and protein needs from breakfast but generally fell well below recommendations for micronutrients.

This aligns with findings from other low-and middle-income countries, where school feeding enhances diet quality but rarely eliminates nutrient gaps. For instance, a study in Ghana reported that beneficiaries of both government and non-government school meal programs still had inadequate total dietary intakes of calcium and vitamin A [22]. A similar pattern was observed in a study from Ghana, where school feeding programs provided sufficient energy and protein but fell short on micronutrient adequacy. Inadequate intake was reported for zinc (35% of beneficiaries), calcium (100%), vitamin B12 (81%), and folate (30%) [23]. A systematic review also concluded that while school feeding effectively reduces hunger and improves diet quality, meal content often remains inadequate to address micronutrient deficiencies without fortification or complementary interventions [24]. These findings underscore the need to enhance school feeding menus not only for diversity but also for nutrient density, including greater use of animal-source foods and fortified staples to address persistent micronutrients gaps.

Table 4 shows that the students’ actual nutrient intake from the ProGAS breakfast was lower than the nutrient content specified in the menu. This may be due to inadequate portioning during food distribution by the staff in-charge, who were generally teachers. Qualitative findings indicated that some students received smaller portions, for example, when unwell or when they requested smaller servings because they were already full. However, in the absence of monitoring by health center staff or the DEO, this explanation could not be verified.

Another possible reason is that students did not finish their meals, as reported by 26.7% of students, partly due to student’s low acceptance of certain menu and limited teacher supervision or encouragement. Low food acceptance is a common challenge in school feeding programs, often linked to poor taste, unappealing presentation, large portions, or unfamiliar ingredients, leading to leftovers such as rice, vegetables, or protein sources [25]. In contrast, Japan’s School Lunch Program reported only 6.9% food waste in 2014. Finishing the meal suggests adequate food consumption, though not necessarily nutrient adequacy. The 2024 Global Survey on School Meal Programs found that 60% of programs consider student food preferences when developing menus [26]. However, improving diet quality may require some restrictions to promote healthier choices, and uniform menus can create social pressure for students to finish their meals. Routine monitoring is also essential, as suboptimal oversight, often due to limited resources and involvement of multiple stakeholders, remains a common global challenge.

In this present study, interviews with the DEO revealed that menu modification guidelines varied across districts. While respecting the ProGAS intention to promote local foods, some districts allowed schools to modify unpopular ProGAS menus, provided the energy and nutrient contents matched the original design, under the supervision of local health center nutritionists. In addition to food acceptance issue, some students did not finish their meals because they had already eaten breakfast at home. As ProGAS meals were typically served at 9–10 a.m., parents were concerned that children would be too hungry if they waited. Considering this issue and the recommendation that breakfast be consumed before 9 a.m., it is important to ensure that the meals are served earlier than 9 a.m.

### 4.2. Pillar 2: Nutrition Education

Low energy and nutrient intakes across the full day (Table 2) and skipping breakfast among children suggest that home dietary practices remain suboptimal. The low dietary intakes among school-aged children in Indonesia have also been documented in the 2019–2020 national nutrition survey, which reported that the proportions of children not meeting Indonesian RNI were 84.9% for energy, 66.5% for protein, 97.8% for calcium, 65.0% for iron, 71.4% for zinc, 82.6% for vitamin A, and 86.1% for vitamin B1 [27].

Although students’ nutrition knowledge improved, it may not yet have translated into behavior change at home, suggesting that parents lack sufficient nutrition knowledge and skills. While participation in cooking committees likely enhanced parental understanding, only a small proportion of parents were involved. Although interest in ProGAS was increasing, many parents might still have lacked the capacity to prepare nutrient-dense meals at home. Therefore, parental nutrition education should be integrated into school meal programs to reinforce healthy practices at home and school [28], with tailored strategies to address the existing challenges such as teachers’ limited nutrition knowledge, workload, inadequate learning materials, and restricted time.

Low energy and nutrient intakes among students may also reflect their socio-economic conditions, as most come from low-resource, food-insecure households. This aligns with the objectives of many school meal programs worldwide, 65% of which serve as social safety nets for vulnerable populations [26]. To address economic constraints that limit dietary improvement, complementary social protection measures, such as conditional cash transfers or food baskets including affordable animal-source foods, should be considered.

This study findings are different from a recent school lunch program in an Indonesian Islamic school that shows improved not only students’ nutrition knowledge and attitudes, but also practices [29]. However, that study assessed micronutrient adequacy using only iron and vitamin C, whereas ProGAS provided a more analysis across multiple nutrients.

### 4.3. Pillar 3: Character Building and Personal Hygiene Practices

In contrast to the implementation of nutrition education, the third component of ProGAS, Character Education, was well integrated into the breakfast session and functioned as ’a prerequisite’ for receiving the meal. These practices included washing hands before and after eating, queuing for handwashing and food distribution, and praying before and after meals. This character development occurred naturally alongside the breakfast routine and became clearly observable, as reported by students and confirmed through direct observations by teachers and school principals. To sustain these good practices, continuous mentoring and stronger monitoring systems are essential [26].

### 4.4. Nutritional Status and Learning Environment Outcomes

In terms of nutritional status, this study did not observe significant changes in BMI-for-age z-scores (BAZ). This finding is consistent with evidence from several low-middle income countries settings where gains in dietary diversity did not immediately translate into anthropometric improvements. Study in Ghana reported that school feeding increased dietary diversity and reduced daily hunger but had no short-term effect on growth indicators [30]. Similarly, evaluations of India’s midday meal scheme show that impacts on anthropometry emerged only after sustained multi-year exposure [31]. These patterns highlight the complexity of translating dietary improvements into measurable growth outcomes. Moreover, nutritional status is shaped not only by diet quality but also by infection burden, household food security, and cumulative exposure to adequate nutrition. Thus, meaningful anthropometric gains may require longer program duration and integration with broader health, sanitation, and social protection strategies [24].

### 4.5. Study Strengths and Limitations

This study evaluates Indonesia’s national school meal program using a mixed-methods approach, providing comprehensive insights. Another key strength lies in the data quality assurance procedures, including data collection by trained enumerators, the use of calibrated tools, identification of under- and over-reporters of energy intake, and the application of the multiple-pass technique for 24 h dietary recall interviews. These efforts may minimize potential bias for 24 h dietary recall among younger children. However, given time and budget constraints, the study did not include a control group, which is acknowledged as a limitation.

## 5. Conclusions

ProGAS led to significant improvements in DDS, particularly in the frequency of egg, fish, and fruit consumption; however, no significant changes were observed in macro- and micronutrient intakes. The program enhanced nutrition knowledge, personal hygiene practices, positive character development, and the learning environment, although breakfast habits remained unchanged at endline. Furthermore, ProGAS did not yield significant improvements in nutritional status, as measured by BMI-for-age z-scores and anemia prevalence. Key management gaps include the delivery of nutrition education to students and the monitoring of implementation by local and national authorities.

## 6. Recommendation

Improvements in eating habits (e.g., meal frequency and DDS) without corresponding increases in nutrient intake highlight the need to strengthen school feeding menus not only for dietary diversity but also for nutrient density, for example, by increasing portions of animal-source foods and incorporating fortified staples. The lower actual nutrient intake from ProGAS breakfasts compared with the planned menu content further indicates the need to enhance the capacity of cooking committees and teachers in portioning, monitoring children’s food consumption, and encouraging meal completion. Clear guidance is also required for menu adjustments in response to food acceptance issues.

Low one-day nutrient intake beyond the ProGAS meal underscores the importance of involving parents in nutrition education to support adequate intake throughout the day, while also identifying families with limited resources who may need assistance beyond education alone to adopt recommended dietary practices. To ensure effective nutrition education delivery, teachers require improved training, interactive IEC materials tailored to school-aged children, and follow-up mentoring. In addition, structured monitoring is essential to promptly identify challenges and document good practices during program implementation.

For future research, using a control group where feasible would strengthen causal inference, and data collection should be conducted while all participants are still receiving the school meals to better capture program effects.

## Figures and Tables

**Table 1:** Students’ socio-demographic characteristics (*n* = 454).

Students’ Socio-Demographic Characteristics	*n* (%)
Sex	
Boys	247 (54.5)
Girls	207 (45.6)
Age in years at baseline (mean, min-max)	10 (8–14)
Age in years at endline (mean, min-max)	11 (9–15)
Father’s education	
No schooling/Completed elementary school	94 (20.7)
Completed junior high school	74 (16.3)
Completed senior high school	148 (32.6)
Completed diploma/bachelor’s degree	51 (11.2)
Do not know	87 (19.2)
Father’s occupation	
Farmers	176 (38.9)
Private employee	68 (15.0)
Government employee	66 (14.6)
Laborer/Construction worker	56 (12.4)
Others	46 (11.1)
Mother’s occupation	
Farmers	172 (38.1)
Housewives	158 (35.0)
Government employee	57 (12.6)
Private employee	19 (4.2)
Laborer	16 (3.5)
Number of siblings	
0–2	237 (52.2)
3 and more	215 (47.8)

**Table 2:** Consumption of food groups among students at baseline and endline of the ProGAS (*n* = 454).

Food Groups Consumed	Baseline *n* (%)	Endline *n* (%)	Delta (%)
Grains, white roots, tubers, plantains	99.8	99.2	−0.6
Legumes (beans, peas, lentils, nuts and seeds)	22.5	25.1	2.6
Milk and milk products	12.1	13.6	1.5
Meat, poultry and fish			
Meat, poultry	22.0	24.0	2
Organ meats	1.1	1.0	−0.1
Fish and seafood	66.5	75.2	8.7
Eggs	28.0	35.8	7.8
Vitamin A-rich vegetables and fruits			
Dark green leafy vegetables	79.1	77.8	−1.3
Yellow fruits	4.6	8.5	3.9
Other fruits and vegetables			
Yellow/orange vegetables	8.0	11.7	3.7
Other fruits	17.2	24.5	7.3

**Table 3:** Dietary intake of students at baseline and endline of the ProGAS Program (*n* = 454).

Median Nutrient ^1^	Sex	Indonesian RNI	EAR	Baseline	Endline	*p* ^2^
Energy (kcal)	Boys	2100	-	1305 (1106–1624)	1419 (1033–1793)	0.517
	Girls	2100	-	1394 (1043–1860)	1402 (1081–1888)	0.876
Carbohydrates (g)	Boys	289	-	218 (177–258)	238 (161–276)	0.478
	Girls	275	-	199 (157–267)	217 (143–250)	0.821
Protein (g)	Boys	56	-	35 (27–50)	44 (34–55)	0.125
	Girls	60	-	44 (28–62)	43 (30–53)	0.520
Fat (g)	Boys	70	-	24 (18–49)	28 (23–47)	0.360
	Girls	67	-	32 (23–56)	32 (27–48)	0.217
Vitamin A (mcg)	Boys	600	429	613 (298–1527)	730 (325–1124)	0.600
	Girls	600	429	670 (357–1513)	675 (477–1085)	0.455
Vitamin B1 (mg)	Boys	1.1	0.9	0.4 (0.3–0.6)	0.4 (0.3–0.6)	0.795
	Girls	1.0	0.8	0.5 (0.3–0.8)	0.4 (0.3–0.7)	0.266
Vitamin B6 (mg)	Boys	1.3	1.1	0.7 (0.6–1.3)	1.0 (0.8–1.4)	0.280
	Girls	1.2	1.0	1.0 (0.7–1.6)	1.0 (0.8–1.4)	0.848
Vitamin B12 (mg)	Boys	1.3	1.1	1.6 (0.8–2.2)	1.5 (1.1–2.8)	0.318
	Girls	1.2	1.0	1.6 (0.9–2.9)	1.6 (0.7–2.4)	0.689
Calcium (mg)	Boys	1200	1000	114 (59–213)	151 (82–243)	0.090
	Girls	1200	1000	163 (94–278)	141 (108–280)	0.958
Iron (mg)	Boys	13	9.3	3.6 (2.6–5.7)	4.8 (3.3–6.1)	0.344
	Girls	20	12.5	4.1 (3.1–5.8)	4.5 (3.4–7.1)	0.305
Zinc (mg)	Boys	14	11.6	3.7 (2.9–4.9)	4.3 (3.4–5.5)	0.185
	Girls	13	10.8	4.2 (3.0–5.2)	4.4 (3.0–6.0)	0.455

^1^ Median of nutrient intakes is presented by 25th–75th percentile. ^2^ Wilcoxon signed rank test.

**Table 4:** Contribution of energy and nutrient intakes from ProGAS breakfast (*n* = 51).

Nutrients	Energy and Nutrient Requirements from Breakfast (25–30% of Total Daily Requirements) *	Intake from the ProGAS Breakfast	
		Median	% Contribution to the Daily Recommendation *^
Energy (kcal)	513–615	355 (210–476)	17.3
Carbohydrate (g)	71–85	57.2 (32.5–87.0)	20.3
Protein (g)	15–17	8.2 (4.0–12.0)	14.1
Fat (g)	17–21	6.2 (2.4–15.4)	9.1
Vitamin A (µg)	150–180	116.7 (6.0–234)	7.8
Vitamin B1 (mg)	0.26–0.32	0.1 (0.05–0.1)	9.5
Vitamin B6 (mg)	0.3–0.4	0.2 (0.1–0.3)	8.0
Vitamin B12 (mg)	0.45–0.55	0.1 (0–0.4)	5.6
Calcium (mg)	300–360	18 (7.7–50.3)	1.5
Iron (mg)	2.9–3.5	0.9 (0.4–1.7)	7.8
Zinc (mg)	3.4–4.1	0.9 (0.5–1.3)	6.7

* Indonesian RNI for children aged 10–12 years [21]). ^ The recommendation for breakfast intake is 25–30% to the daily requirements.

**Table 5:** Nutrition knowledge among students at baseline and endline (*n* = 454).

Knowledge Question to be Answered Correctly	Correct Answer	Baseline %	Endline %	Δ %
Water is important for us. If there is a lack of water consumption, the body will become weak	True	82.4	87.6	5.2
2. Before eating we should wash our hands even if our hands are not dirty	True	87.1	91.4	4.3
3. By only washing your hands with water is enough.	False	59.2	77.3	18.1
4. Brushing your teeth before bed can prevent cavities.	True	79.0	84.1	5.1
5. Cleaning the classroom is one of the physical activities.	True	84.5	86.5	2.0
6. To know that we are growing well, we need to measure our weight regularly.	True	90.1	91.4	1.3
7. Breakfast should be done before 9 am.	True	76.1	74.8	−1.2
8. Not having breakfast will make us unable to study well in class.	True	69.9	75.9	6.0
9. Breakfast with sweet tea is enough.	False	41.5	64.5	23.0
10. If we eat less than 3× a day, the body will become weak and not powerful.	True	72.4	76.4	4.0
11. Eating 3× a day causes the body to become obese.	False	30.5	36.4	5.9
12. Fish and eggs are sources of building blocks or protein.	True	86.3	94.0	7.7
13. Rice, corn, cassava, and sweet potatoes are sources of energy.	True	87.3	90.9	3.6
14. Vegetables and fruits are a good source of vitamins so that we do not easily get sick.	True	89.5	94.5	5.0
15. We should eat vegetables at least 3× a day	True	77.7	71.3	−6.4

**Table 6:** Character building and personal hygiene practices among students at baseline and endline (*n* = 454).

Practice	Baseline, *n* (%)	Endline, *n* (%)	*p*-Value ^1^
Character building practices			
Bringing drinking water to school (always/often)	177 (39.2)	204 (45.1)	<0.001
Praying at the beginning of lessons (always/often)	225 (49.7)	277 (61.3)	<0.001
Praying before meals (always/often)	192 (42.3)	266 (58.8)	<0.001
Queuing (always/often)	273 (60.1)	286 (63.1)	<0.001
Finishing meals (always/often)	32 (7.0)	33 (7.3)	<0.001
Personal hygiene practices			
Washing hands with soap before eating	392 (86.3)	436 (96.2)	<0.001
Washing hands in 5 critical times	208 (45.8)	237 (52.3)	0.093
Brushing teeth more than twice a day	139 (30.6)	210 (46.5)	<0.001
Taking a bath twice a day	259 (57.0)	283 (62.7)	0.012
Cleanliness of nail, short and clean	176 (38.8)	234 (52.0)	<0.001
Cleanliness of hair, skin looks clean	300 (66.2)	347 (76.4)	<0.001

^1^ McNemar Test.

**Table 7:** Percentage of students’ performance at baseline and endline (*n* = 454).

Students’ Performance in Class	Baseline *n* (%)	Endline *n* (%)	*p*-Value ^1^
Active in class	222 (49.2)	238 (52.7)	0.369
Feeling hungry during class	329 (72.4)	290 (64.6)	0.008
Feeling sleepy during class	231 (50.9)	219 (48.3)	0.425
Feeling comfortable at class	336 (74.2)	397 (87.6)	<0.001

^1^ McNemar Test.

**Table 8:** Benefits and Challenges of ProGAS Implementation Perceived by Various Stakeholders.

Aspect	Perceived Benefits	Examples of Quotes
Students’ nutrient requirement	○ Fulfilled students’ energy and nutrient requirements through breakfast, particularly for those from low-income families, those who did not have time for breakfast at home, or whose parents were unable to prepare meals due to work	*“Children from low-income families are greatly helped by receiving additional nutritious food served by the program. It in turn affects their learning process.” (School Principal)* *“Usually, my child has breakfast at home. But if I am busy, then they don’t. (After ProGAS) we are happy, because even if they don’t have breakfast at home, we are not worried, as they stay at school until 2 p.m.” (Parents)*
Students’ dietary and hygiene practices	○ Improved students’ breakfast habits after ProGAS, with greater willingness to eat breakfast, as well as to consume vegetables, fruit, and fish ○ Students showed improved personal hygiene practices, and therefore, looked cleaner and neater	*“It is better, because now they want to eat vegetables. The school provides nutritious food and fruit, which is previously rarely consumed at home.” (Parents)* *“Children became healthier, do not get sick easily. They also have noticeable improvements in personal hygiene, such as brushing their teeth more regularly, bathing more frequently, and trimming their nails more diligently.” (Parents)*
Students’ learning environment and participation	○ Increased student attendance ○ Greater enthusiasm for learning, with students able to stay engaged until lessons concluded ○ Higher new student enrolment in the new academic year	*“Overall, student attendance has increased. It seems that students do not want to miss the opportunity for breakfast, so they come to school more regularly. (School Principal)*
Students’ character building	○ Improved handwashing habits ○ Improved habits of bringing drinking water from home ○ Greater gratitude through praying ○ Better discipline and independent during meals (e.g., sitting properly, finishing food, cleaning meal box and utensils) ○ Increased initiative to lead ○ Enhanced helpfulness and respect toward peers	*“They used to pray before and after class only. Now they also pray before and after eating. To show gratitude.” (Teacher)*
Parents’ involvement	○ Parents showed increased participation in school committee meetings	*“They are very happy, often hearing stories from their children and sometimes asking about the ProGAS menu that day.” (School Principal)* *“Parents regularly come to school. They are pleased because their farm produce can be sold to the school without having to go all the way to the market. Parents are also involved as part of the cooking team.” (School Principal)* *“Parents can also build closer communication with the school and learn what healthy menus look like. (Parent)*
Community learning and income earning	○ Learning to cook healthy and safe meals, particularly incorporating vegetables into children’s menus, and applying it at home ○ Earning additional income for savings, primarily allocated to children’s needs, daily household expenses, special occasions such as Eid and New Year, and small business capital	*“Now I can cook two new healthy menus: fried noodles with corn and vegetables, and boiled cassava with vegetables and tofu-egg.” (Cooking Team)* *“I also learned from the ProGAS menus and got the idea to disguise vegetables, so after ProGAS, my child’s meals have included more vegetables.”* *(Parents)*
Collaboration and communication among stakeholders	○ Teachers and principals shared their feedback and photos through the WhatsApp group of principals and the district education office	
Others	○ Reduced household spending on students’ pocket money	*“My child used to be a picky eater, only wanting pocket money and refusing breakfast. (After ProGAS) they have started to enjoy breakfast and no longer ask for pocket money, saying: ‘Mama, no need to give me pocket money, we eat at school.” (Parent)*

## Data Availability

The data presented in this study are available on request from the corresponding author due to data ownership held by the Ministry of Primary and Secondary Education, and sharing requires prior authorization from the Ministry.

## Abbreviations

The following abbreviations are used in this manuscript:

BAZ	BMI-for-age z-scores
BMI	Body Mass Index
DDS	Dietary Diversity Score
DEO	District Education Office
EAR	Estimated Average Requirement
ProGAS	Program Gizi Anak Sekolah (School Feeding Program)
RNI	Recommended Nutrient Intake
