# Correction: Pramesthi et al. Evaluating the Impact of Indonesia’s National School Feeding Program (ProGAS) on Children’s Nutrition and Learning Environment: A Mixed-Methods Approach. *Nutrients* 2025, *17*, 3575

**DOI:** 10.3390/nu18121938

**Published:** 2026-06-16

**Authors:** Indriya Laras Pramesthi, Luh Ade Ari Wiradnyani, Roselynne Anggraini, Judhiastuty Februhartanty, Wowon Widaryat, Bambang Hadi Waluyo, Agung Tri Wahyunto, Muchtaruddin Mansyur, Umi Fahmida

**Affiliations:** 1 Southeast Asian Ministers of Education Organization Regional Centre for Food and Nutrition (SEAMEO RECFON), Pusat Kajian Gizi Regional (PKGR) Universitas Indonesia, Jakarta 13120, Indonesia; 2 Department of Nutrition, Faculty of Medicine, Universitas Indonesia–Dr. Cipto Mangunkusumo General Hospital, Jakarta 10430, Indonesia; 3 Department of Nutrition, Faculty of Sports and Health Sciences, Universitas Negeri Surabaya, Surabaya 60231, Indonesia; 4 Directorate General of Primary and Secondary Education, Ministry of Education and Culture Republic of Indonesia, Jakarta 10270, Indonesia; 5 Department of Community Medicine, Faculty of Medicine, Universitas Indonesia, Jakarta 10310, Indonesia; muchtaruddin.mansyur@gmail.com

There was an error in the original publication [1] regarding one reference cited in Section 4.1. After publication, the authors realized that the findings from Ghana were incorrectly attributed to Gelli et al. (2019). The correct source should be Abizari et al. (2014) and listed in the reference list as:

23. Abizari, A.-R.; Buxton, C.; Kwara, L.; Mensah-Homiah, J.; Armar-Klemesu, M.; Brouwer, I.D. School feeding contributes to micronutrient adequacy of Ghanaian schoolchildren. *Br. J. Nutr.* **2014**, *112*, 1019–1033. https://doi.org/10.1017/S0007114514001585.

This correction does not affect the results, interpretation, or conclusions of the article.

A correction has been made to Section 4.1 by updating Reference No. 23 in the reference list. No changes to the discussion narrative were required. The authors state that the scientific conclusions are unaffected. This correction was approved by the Academic Editor. The original publication has also been updated.

## References

[1] Pramesthi I.L., Wiradnyani L.A.A., Anggraini R., Februhartanty J., Widaryat W., Waluyo B.H., Wahyunto A.T., Mansyur M., Fahmida U. (2025). Evaluating the Impact of Indonesia’s National School Feeding Program (ProGAS) on Children’s Nutrition and Learning Environment: A Mixed-Methods Approach. Nutrients.

